# Supplementary material for: Mechanism and modulation of terahertz generation from a semimetal - graphite
Source: Sci Rep. 2016 Mar 14;6:22798. doi: 10.1038/srep22798 (PMC4789595; doi:10.1038/srep22798)
Supplement: Supplementary Information [file srep22798-s1.doc]

Mechanism and modulation of terahertz generation from a semimetal - graphite

Tong Ye^1^, Sheng Meng^1^, Jin Zhang^1^, Yiwen E^1^, Yuping Yang^2^, Wuming Liu^1^, Yan Yin^1,3,*^, Li Wang^1,3^

1, Beijing National Laboratory for Condensed Matter Physics, Institute of Physics, Chinese Academy of Sciences, Beijing 100190

*2, School of Science, Minzu University of China, Beijing, 100081*

*3, Cooperative Innovation Centre of Terahertz Science,* *Chengdu, Sichuan, 610054*

*Email address: yan.yin@aphy.iphy.ac.cn

**Supplementary information:**

1. About the dispersion of the value of *I_p_*/*I_n_*.

In order to check the dispersion of the value of *I_p_*/*I_n_*, we performed a second measurement more than half year after the first measurement (from which we got data in the figure 2 of the main text). In our second measurement, we used a different, but same kind graphite sample. Both samples are ZYA grade HOPG graphite from Structure Probe Inc. This second measurement’s results are plotted in Figure 1, and give an *I_p_*/*I_n_*=2.2. Together with the *I_p_*/*I_n_*=2.3 from the first measurement, we have
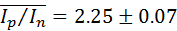
 for good quality HOPG samples.


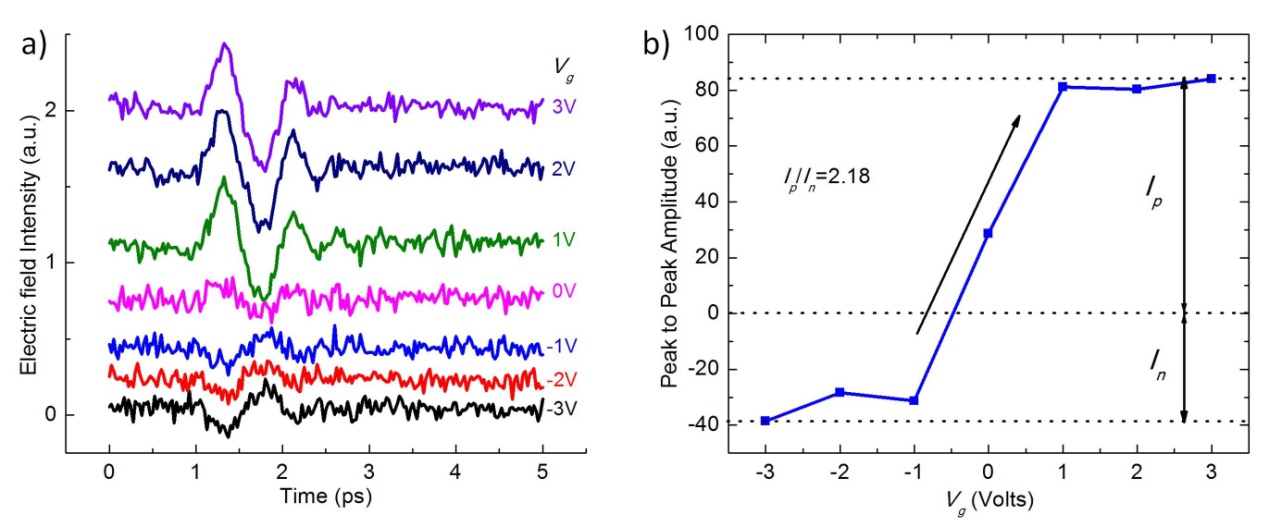


**Figure 1 | (a) THz waveforms and (b) peak-to-peak amplitudes as functions of the gate voltage (*V_g_*) of measurement 2.** In sub-figure (a), difference color curves represent the THz waveforms from graphite under different *V_g_* (corresponding values listed on the right side). In sub-figure (b), the data points are from *V_g_* upward scan.

2. Spectral data for the THz generation from graphite surface.

Figure 2 shows the spectral data of the THz generation from graphite surface. There are two measurements; both measurements are under similar conditions, but with two different, same kind HOPG samples. Waveforms from measurement 1 are plotted in Figure 2 of the main text, and waveforms from measurement 2 are plotted in Figure 1 of this supplementary information file.

Figure 2(a) shows the spectral data for different gating voltage *V_g_* from measurement 1. The legends indicate the *V_g_*, used in the measurements. From the data, we can see that the THz radiation from the graphite increases with a higher gating voltage *V_g_*. The positive *V_g_* side shows a much stronger saturation signal level than the negative *V_g_* side. It seems that the spectral data under *V_g_* bias has two peaked line-shape. The physical origin of this two peak line-shape is unknown. Figure 2(b) shows the spectral data for two different measurements at the max signal strengths. The two measurements show different spectral line-shape; the underlying reason is not clear.

For the spectral bandwidth, we consider the strongest signal spectrum which is under *V_g_*=2.5V in measurement 1; the half maximum locations are about 0.5THz and 2.2THz, this gives us a spectral bandwidth about 1.7THz. Because the 0.5THz end is very close to the end of the response range of the system, the true bandwidth might be slightly higher than 1.7THz. Therefore, the spectral bandwidth should be about 1.7THz, with possibility up to 2.2THz.

In comparison to the spectral bandwidth of semiconductor THz emitters, graphite falls in the middle. Some of the semiconductors have broader; some have narrower spectral bandwidth than that of graphite. The predicted very broad spectral bandwidth of graphene emitters due to graphene’s high carrier mobility, doesn’t apply to here. The prime physical reason of THz generation from graphite is the carrier movements in Z direction, which have a couple orders of magnitude smaller carrier mobility than that of in-plane movements in graphene.


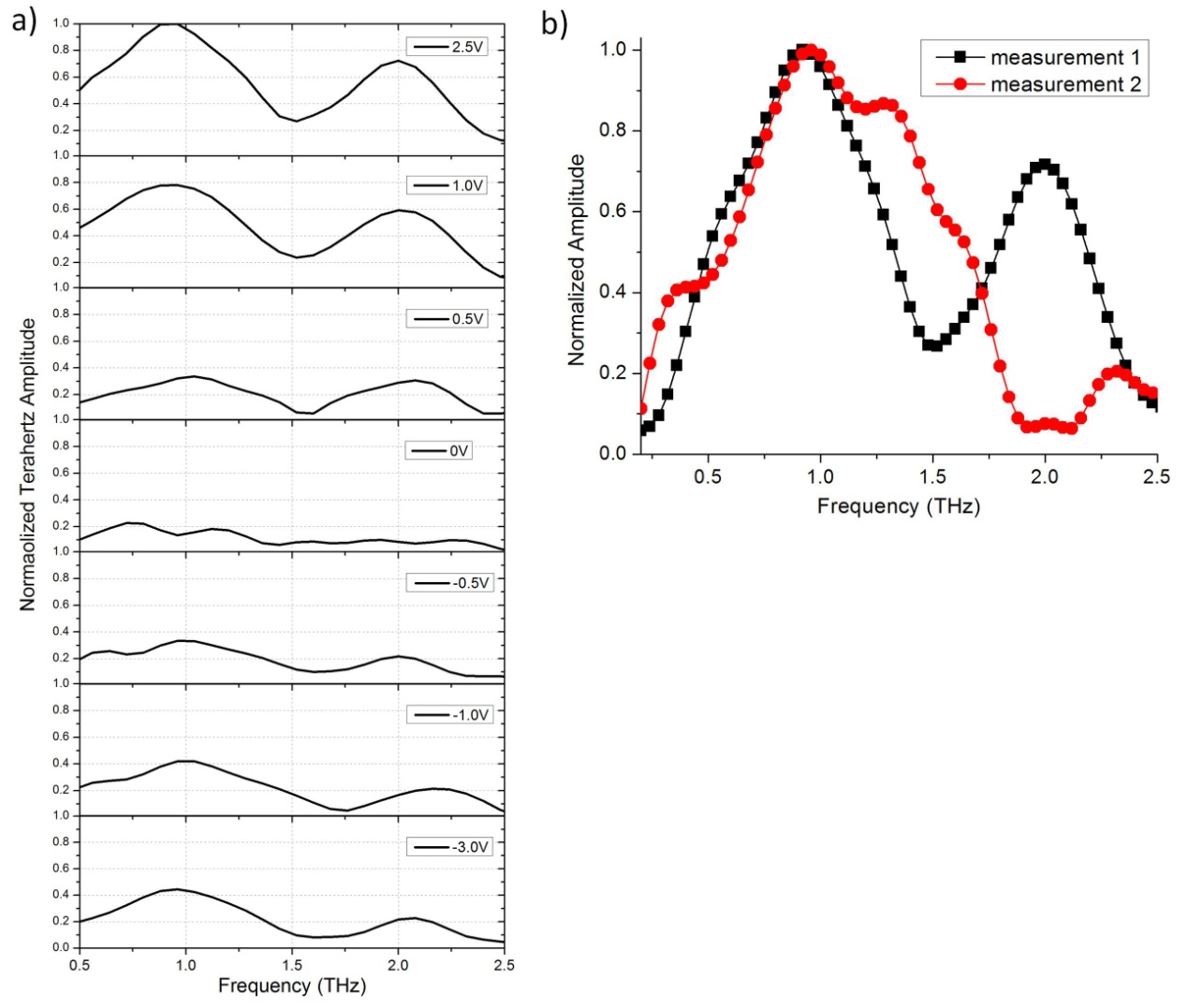


**Figure 2 | The spectral data of the THz generation from graphite surface.** (a) For different gating voltage *V_g_* from measurement 1; (b) for measurement 1 and measurement 2 at the max signal strengths. In sub-figure (b), the black square data points are from measurement 1 when *V_g_*=2.5V; the red dot data points are from measurement 2 when *V_g_*=3V.

3. Discussion about the effective masses.

In our main text, we give out the effective masses in Z direction of the surface of graphite when the Fermi surface is at ±0.2eV. Our values are 5.5m_0_ and 2.5m_0_. The generally reported effective mass of graphite is about 0.045m_0_ (from calculation) and 0.06m_0_ (from experiment); those general values are really only for the quasi-particle within the XY plane (the basal plane). The large difference between our values and the “general” values is mainly due to the directional difference between Z direction and XY plane directions. Such a large difference is reasonable, because the electrical conductivity within XY plane is 4000 times of the electrical conductivity in Z direction. Also, we would like to point out that our values are for Fermi surface at ±0.2eV; should Fermi surface change, the effective mass will change too.
